# Awareness among Pregnant Women about Epidural Analgesia: A Cross-Sectional Study

**DOI:** 10.1155/2022/7388833

**Published:** 2022-06-10

**Authors:** Waad M. Almuntashiri, Aseel S. Mutawakkil, Amjad S. Alghamdi, Razan D. Alqarni, Alaa M. Althubaiti, Haifaa S. Kayal

**Affiliations:** ^1^College of Medicine, King Saud Bin Abdulaziz University for Health Sciences, Jeddah, Saudi Arabia; ^2^King Abdullah International Medical Research Center, Jeddah, Saudi Arabia; ^3^Department of Anesthesiology, King Abdulaziz Medical City, Ministry of the National Guard Health Affairs, Jeddah, Saudi Arabia

## Abstract

**Background:**

Epidural analgesia (EPA) is an effective anesthetic technique to overcome pain during labor. This study aimed to evaluate the current awareness of EPA among pregnant women.

**Methods:**

We carried out a cross-sectional study using a questionnaire to measure awareness about EPA among pregnant women visiting the obstetrics and gynaecology clinic in King Abdulaziz Medical City in Jeddah. Following the results, a group of women was selected and educated by trained medical students.

**Results:**

This study comprised 105 women. We found that 25 (23.8%) respondents revealed a complete lack of knowledge regarding EPA, 63 (60%) showed minimal awareness, and 17 (16.2%) were aware of EPA from various sources. The gravidity and history of EPA administration were significantly associated with knowledge. Multigravida women and those who had received EPA showed higher level of knowledge (*p*=0.048 and *p* < 0.001, respectively). In addition, there was a significant association between the level of education and request for EPA (*p*=0.027). Forty-one participants were enrolled in an educational program that explained the importance of EPA. Twenty (48.8%) women decided to undergo EPA during delivery; however, 7 (17.7%) refused and 14 (34.1%) were not sure about their decision.

**Conclusion:**

This study revealed a lack of awareness about EPA among pregnant women. Educational programs were effective as many participants chose EPA following the educational session as a form of pain relief during labor. We recommend the implementation of routine education on EPA in vaginal delivery during antenatal visits for all pregnant women.

## 1. Introduction

Childbirth is a complicated experience that leaves mothers in a paradoxical state where they are overwhelmed by a sensation of happiness, fear, and severe pain. Labor pain is a multifactorial event in which pregnant women experience physiological, psychological, and psychosocial distress. Different interventions can be implemented to facilitate labor.

Epidural analgesia (EPA) is an effective anesthetic technique to overcome the hardship of labor [1]. Epidural analgesia is a rapid approach for pain management during parturition. The procedure is conducted via a needle inserted in the epidural space covering the spinal cord. This method produces a full sensory block in the lower region of the abdomen and lower limbs [2]. The onset of pain relief begins 20 to 30 minutes after the administration. The effect of the drug lasts for several hours before it starts to wear off [3]. Even though it is useful and simple, many women refrain from receiving EPA during labor due to lack of awareness.

Some medical centers fail to provide appropriate antenatal education regarding EPA. Moreover, in some medical facilities, women are provided with new information and are required to make quick and significant intrapartum decisions, thus resulting in a negative birth experience [4].

The Obstetric Anesthetists' Association recommends that the anesthetic department should provide all expectant mothers with up-to-date information on analgesia regardless of their birthing plan for early labor in pregnancy [5]. According to a recent study conducted in London, of 742 women preparing for vaginal delivery, 67 (9%) recalled receiving antenatal information covering all aspects of labor analgesia and 127 (17%) did not recall receiving any information [6].

To the best of our knowledge, a study was conducted in Jeddah in 2017 to investigate the awareness of EPA among pregnant women. Another study carried out in Riyadh in 2018 focused on evaluating the total awareness of EPA among primigravida women and the factors contributing to their decision. An educational program was also conducted to understand the advantages of a health education program regarding EPA. These results indicate the necessity of education regarding EPA for expectant mothers [7, 8].

Due to the insufficiency of research and education regarding EPA, we aimed to evaluate the current awareness of EPA among pregnant women. In addition, we sought to conduct an educational program for primigravida and multigravida women who are in their third trimester and guide them toward proper epidural education.

## 2. Methods

We carried out a cross-sectional study among pregnant women in King Abdulaziz Medical City (KAMC), Jeddah, Saudi Arabia (a tertiary care center). We selected the participants from the obstetrics and gynaecology department, which has about six beds for active delivery and handles approximately 3114 deliveries per year, of which 33.1% (1031) are by cesarean section and 60.5% (1884) are normal spontaneous deliveries. We included pregnant women in their third trimester who planned to undergo vaginal delivery and excluded those who had to undergo cesarean delivery. We calculated the sample size using Raosoft online calculator as 274 pregnant women from a population of 942 women (in Jeddah) assuming a confidence interval of 95% and margin of error of 5%. In the sampling technique, we distributed a questionnaire in the OB/GYN clinic in KAMC after obtaining consent of the participants using a consent form prior to the questionnaire. The questionnaire was validated by a previous study conducted by Alakeely [8], and permission was obtained to utilize the survey. Trained medical students supervised by a consultant in the anesthetic department collected the data from the questionnaire. The questions were tested on a pilot sample of five pregnant women. For the educational session, a group of women was selected to undergo an educational program virtually via phone due to COVID-19 restrictions. Later, we followed up and interviewed the women postpartum to gain knowledge of their decision.

### 2.1. Data Analysis

Frequencies and percentages were used to describe categorical variables (e.g., educational level, employment status, income, residency, and gravidity), and the chi-square or Fisher's exact tests were used to compare them. The statistical significance was set at *p* < 0.05. Statistical analyses were performed using John's Macintosh Project (JMP) version 8.1 (SAS Institute Inc., Cary, NC).

### 2.2. Ethical Consideration

We carried out this study after the approval of the Institutional Review Board, under protocol number SP20/033/J. We obtained participants' consent before distributing the questionnaire.

## 3. Results

### 3.1. Demographic Characteristics

The study comprised 105 women. The mean age was 30 ± 5.7 years (age range, 19 to 41 years). The total number of primigravida pregnancies was 32 (30.5%), and 24 (22.9%) participants had previously received EPA for vaginal delivery (Table 1).

### 3.2. Association between Demographic Characteristics and General Knowledge about Epidural Anesthesia

Of the 105 respondents, 25 (23.8%) revealed a complete lack of knowledge regarding EPA, 63 (60%) showed minimal awareness, and 17 (16.2%) were aware of EPA from various sources. The results showed that age, income, residency, employment status, and level of education were not significantly associated with the knowledge on EPA. Previous exposure to EPA and gravidity were significantly associated with knowledge. Women who received EPA prior to the current pregnancy showed higher level of knowledge than those who did not receive it before (41.7% vs. 8.6%, *P* < 0.001). Multigravida women showed high levels of knowledge regarding EPA (21.9% vs. 3.1%, *P*=0.048).  Table 2 presents the association between demographic characteristics and general knowledge on EPA.

### 3.3. Association between Demographic Characteristics and the Desire to Receive Epidural Anesthesia

The results show that age, income, employment status, residency, number of births, and prior exposure to EPA were not significantly associated with the desire to receive EPA. Education level showed a significant association (*P*=0.027) (Table 3).

### 3.4. Preferred Method to Receive Education on EPA

**Figure 1:**
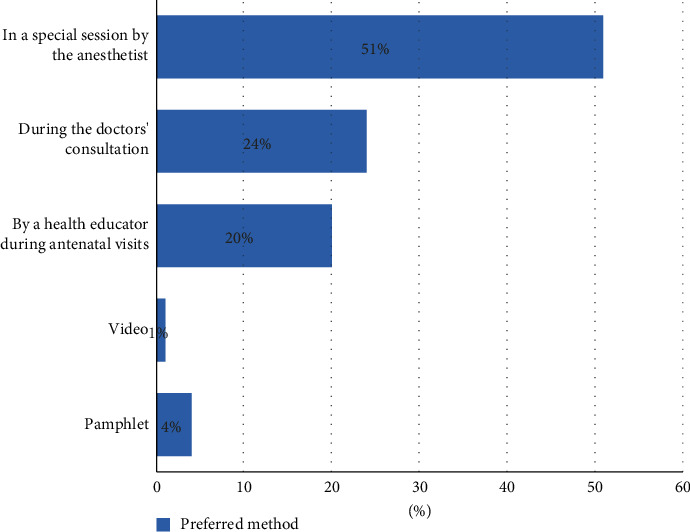
Preferred method for education.

When the participants were asked if they preferred the procedure to be explained during antenatal visits, 89 (85%) answered yes. Those who answered yes were asked a follow-up question about the most suitable way to explain the procedure: 51% preferred receiving education by an anesthetist and 24% chose to receive education during OB/GYN consultation (Figure 1).

### 3.5. Educational Program

Of the 105 participants, 41 agreed to be enrolled in an educational program that explained the importance of EPA. Twenty (48.8%) women decided to undergo EPA for their delivery; however, 7 (17.1%) refused to receive EPA, and 14 (34.1%) were not sure about their decision.

We followed up the 34 participants who either agreed or were not sure about receiving EPA postpartum. Four participants received EPA for vaginal delivery, and the rest of the participants did not receive EPA as seen in the Discussion section.

## 4. Discussion

Our results revealed that 16.2% of the participants were aware of EPA from different sources. We concluded that multigravida participants and women with previous exposure to EPA showed higher levels of knowledge about EPA than those who were primigravida and those who were not previously exposed to EPA. In addition, participants with higher educational levels were more likely to request EPA during labor. Compared to other studies, our study assessed the awareness on EPA and factors associated with the knowledge on EPA. We found that gravidity and previous exposure to EPA were significantly associated with the participants' level of knowledge. A recent study conducted in Khamis Mushait [9] reported findings similar to our results regarding the association between previous exposure and knowledge. A study conducted in India revealed that residency in villages influenced the expectant mothers' knowledge regarding EPA [10], which was inconsistent with our results. Another study found that education, occupation, habitat, previous knowledge regarding EPA, and period of gestation were significantly associated with their knowledge [11].

In addition, we studied the factors that could influence participants' desire to receive EPA during labor and found that women with higher levels of education were more likely to request EPA during delivery, which was consistent with a previous study conducted in Vietnam [12]. Similarly, a study carried out in the United States revealed a significant association between the level of education and epidural request [13]. Factors such as employment were associated with the request of the epidural analgesia in a study conducted in Riyadh [8], which was inconsistent with our results. A couple of studies found that expectant mothers with health insurance were more likely to request EPA during labor [12, 14]. In contrast, in our study, financial factors did not contribute to decision-making because our study setting was in a government hospital where free health care is provided.

In this study, we conducted an educational program on EPA targeting participants in their third trimester, which proved effective as 48.8% decided to receive EPA during delivery. There is a need for education on EPA during antenatal visits. Some studies supported this finding because there is a lack of awareness among pregnant women in many countries, which emphasizes the need for education on EPA [9, 15, 16]. After the education, we followed up the participants who found the program beneficial and decided to undergo EPA postpartum to know whether they had received EPA during delivery and why. Four participants received EPA during delivery and were satisfied with the results. The remaining participants could not receive EPA due to insufficient time for application or complications during labor, as most had to undergo cesarean section for different reasons.

To the best of our knowledge, this is the second study in Jeddah to measure the level of awareness of EPA among pregnant women and the fifth in the country. This is the first study to conduct an educational program in Jeddah and second in the country [8]. In addition, this is the first study that followed up the participants postpartum to measure the effectiveness of education in Saudi Arabia. This study had several limitations. The COVID-19 pandemic restricted the data collection process, which affected the sample size. In addition, the method of conducting the education was changed from in-person sessions to virtual sessions, which limited the number of responses because not every participant was available via phone. Our study was performed in King Abdulaziz Medical City, which probably influenced our results and cannot be generalized to the Saudi Arabian population.

## 5. Conclusion

This study revealed the lack of awareness of EPA among pregnant women, and educational programs proved effective as many participants were willing to use EPA as a form of pain relief during labor. We recommend implementing routine educational sessions on the use of EPA in vaginal delivery during antenatal visits for all pregnant women.

## Figures and Tables

**Table 1:** Demographic characteristics.

Demographic variables	*N* (%)
Age (mean ± SD) years	30 ± 5.7
Level of education	
School-level education	39 (37.1)
University-level education	66 (62.9)
Employment status	
Employed	27 (25.7)
Not employed	78 (74.3)
Family monthly income	
Less than 10,000 SAR	77 (73.3)
More than 10,000 SAR	28 (26.7)
Previous exposure to epidural analgesia	
Yes	24 (22.9)
No	81 (77.2)
First pregnancy	
Yes	32 (30.5)
No	73 (69.5)

Abbreviations: SAR − Saudi riyal; SD = standard deviation.

**Table 2:** Demographic characteristics and general knowledge regarding EPA.

General knowledge regarding epidural analgesia	I know a lot (*n* = 17)	I know a little (*n* = 63)	I do not know (*n* = 25)	*P* value
Level of education				
School-level education	5 (12.8)	21 (53.9)	13 (33.3)	
University-level education	12 (18.2)	42 (63.6)	12 (18.2)	0.75
Employment status				
Employed	6 (22.2)	17 (62.9)	4 (14.8)	0.59
Not employed	11 (14.1)	46 (58.9)	21 (26.9)	
Family monthly income				
Less than 10,000 SAR	11 (14.3)	45 (58.4)	21 (27.3)	0.49
More than 10,000 SAR	6 (21.4)	18 (64.3)	4 (14.3)	
Previous exposure to epidural analgesia				
Yes	10 (41.7)	10 (41.7)	4 (16.7)	<0.001
No	7 (8.6)	53 (65.4)	21 (25.9)	
First pregnancy				
Yes	1 (3.1)	23 (71.9)	8 (25)	0.048
No	16 (21.9)	40 (54.8)	17 (23.3)	

**Table 3:** Demographic characteristics and desire to receive epidural anesthesia.

Epidural analgesia	Yes (*n* = 38)	No (*n* = 30)	Not sure (*n* = 37)	*P* value
Level of education				
School-level education	10 (25.6)	17 (43.6)	12 (30.7)	
University-level education	28 (42.4)	13 (19.7)	25 (37.9)	0.028
Employment statue				
Employed	11 (40.7)	7 (25.9)	9 (3.3)	0.85
Not employed	27 (34.6)	23 (29.5)	28 (35.9)	
Family monthly income				
Less than 10,000 SAR	30 (38.9)	21 (27.3)	26 (33.7)	0.62
More than 10,000 SAR	8 (28.6)	9 (32.1)	11 (39.3)	
Previous exposure to epidural analgesia				
Yes	12 (50.0)	6 (25.0)	6 (25.0)	0.11
No	26 (32.1)	24 (30.8)	31 (39.7)	
First pregnancy				
Yes	10 (31.3)	10 (31.3)	12 (37.5)	0.78
No	28 (38.4)	20 (27.4)	25 (34.3)	

## Data Availability

Data are available on request to the corresponding author.
